# Clinical observations from the clinical video head pulse test in patients with idiopathic horizontal semicircular canal light cupula

**DOI:** 10.3389/fneur.2024.1496430

**Published:** 2024-12-11

**Authors:** Niu Song, Chang Jingling, Xu Wenyan, Pei Xuemei

**Affiliations:** Department of Neurology, Dongzhimen Hospital Affiliated to Beijing University of Chinese Medicine, Beijing, China

**Keywords:** light cupula, direction-changing positional nystagmus, video head impulse test, vestibular function test, cupulolithiasis

## Abstract

**Objective:**

The objective of the study is to analyze and explore the characteristics of the video head impulse test (vHIT) for light cupula in the idiopathic horizontal semicircular canal and compare them with those of horizontal semicircular canal cupulolithiasis (HC-cu) in order to investigate the potential mechanism involved.

**Methods:**

Data from 51 cases of idiopathic light cupula and 42 cases of horizontal semicircular canal cupulolithiasis were retrospectively analyzed. The positional nystagmus features, vHIT anomaly rate, gain value, saccades, and other indicators were compared. SPSS 26 and Medcalc 22 were used to analyze the differences and correlations.

**Results:**

There were no differences in sex, age, the affected side, and positional nystagmus between the light cupula group and HC-cu group (*p* > 0.05). The overall abnormal rate of the vHIT (56.86% vs. 21.43%), the abnormal rate of the affected side (23.53% vs. 0.00%), and the saccade ratio of the affected side [40.0 (13.0, 76.0) vs. 6.0 (0.0, 15.0)] in the light cupula group were higher than those in the HC-cu group. The HC saccade ratio in the light cupula group was higher on the affected side than on the healthy side [40.0 (13.0, 76.0) vs. 11.0 (0.0, 38.0)], and the differences were statistically significant (*p* < 0.05). The receiver operating characteristic (ROC) curve analysis showed that the abnormal vHIT results and saccade ratio of the light cupula group were correlated with the affected side (*p* < 0.05).

**Conclusion:**

The vHIT results of idiopathic HC light cupula and HC-Cu were different as they are distinct diseases. Light cupula may be associated with some mild lesions in the vestibulo-ocular reflex (VOR) pathway. The lighter cupula theory is the possible mechanism.

## 1 Introduction

In recent years, the light cupula (1, 2) with no history of alcohol intake has gained attention as an explanation for direction-changing positional nystagmus (DCPN). The main clinical manifestation of this disease is paroxysmal positional vertigo. Physical examination may reveal geotropic DCPN, a zero plane where the nystagmus disappears (3), and pseudo-spontaneous nystagmus (PSN) (4). Light cupula is not rare, accounting for 14.2% of cases of geotropic DCPN (5), and there is no effective treatment available (6, 7). The exploration of its mechanism and treatment methods remains a key focus for clinicians. Currently, the mechanism of this disease is still controversial, but several hypotheses related to the vestibular system have been proposed (8, 9). Existing imaging techniques and anatomical methods offer limited insights into the mechanism of this disease, while vestibular function examination may help enhance our understanding of it. Previous studies have shown that light cupula can occur as a secondary condition to other inner ear-related diseases, such as sudden deafness (10–12) and labyrinthitis (13). These diseases usually have clear vestibular dysfunction. However, light cupula can also occur in isolation, i.e., idiopathically. Is idiopathic light cupula associated with vestibular dysfunction? This is a question of concern, and it may provide clues for exploring the disease’s mechanisms and therapeutic methods. Few studies have addressed this issue. In this study, the video head impulse test (vHIT) was used to evaluate the function of the high-frequency vestibulo-ocular reflex (VOR) pathway for each semicircular canal (14), and cases of idiopathic horizontal semicircular canal light cupula were analyzed. By comparing idiopathic horizontal semicircular canal light cupula with horizontal semicircular canal cupulolithiasis (HC-cu), this article discusses both the similarities and differences, as well as the related mechanisms. This study aimed to improve clinicians’ understanding of this disease and provide a reference for further exploration into its treatment and prevention. The findings are presented as follows.

## 2 Materials and methods

### 2.1 Sample sources

This study was a retrospective analysis. It was carried out in the Vertigo Center of Dongzhimen Hospital, Beijing University of Chinese Medicine. The research participants were selected from the case database of the vestibular function examination room, covering the period from 1 January 2021 to 31 December 2023. The vestibular examination room in our center, established in 2015, includes a complete case database. The database used in this study was sourced from the existing databases. Based on the pre-test results of the abnormal rates of the vHIT in the two groups, with a significance level of 0.05, a test power of 0.8, and a grouping ratio of 1:1, the sample size was calculated. The sampling method involved retrospectively extracting all cases that met the inclusion criteria within a specific time period. We ensured data quality by establishing rigorous data collection standards, training professional staff, conducting double-entry verification, performing comprehensive data cleaning and auditing, and applying multi-method cross-validation.

According to the inclusion/exclusion criteria, the cases were divided into the light cupula group and the HC-cu group. As only a small fraction of typical HC-cu cases had vHIT results and the total number of idiopathic light cupula cases was limited, all eligible cases during the review period were included in this study to increase the sample size.

This study was approved by the Medical Ethics Committee of Dongzhimen Hospital (2022DZMEC-251), and a waiver for informed consent was obtained.

### 2.2 Inclusion and exclusion criteria

The inclusion criteria for the light cupula group were as follows: (a) age ranging from 18 to 80 years. (b) The roll test showing persistent geotropic DCPN with no significant latency and attenuation, and the duration of nystagmus being greater than 1 min. The roll test identifying null plane 2 (NP2) (3), and the Bow and Lean test (BLT) (15) being positive. (c) Brain imaging (MRI/CT) and a nervous system examination excluding central lesions.

The inclusion criteria for the HC-cu group were as follows: (a) age ranging from 18 to 80 years. (b) The diagnostic criteria following the benign paroxysmal positional vertigo (BPPV) diagnostic criteria for horizontal semicircular canal cupulolithiasis (16), i.e., recurrent vertigo attacks caused by lying down or turning over, with the roll test showing continuous apogeotropic DCPN with a duration of >1 min, which could not be attributed to other diseases.

The exclusion criteria were as follows: (a) coexisting vestibular disorders, such as vestibular migraine, Meniere’s disease, vestibular neuritis, sudden deafness with vertigo, and typical BPPV; (b) a history of alcohol intake within the past 48 h; (c) use of vestibular inhibitors within the past 72 h; and (d) presence of central lesions, posterior circulation ischemia, psychosis, epilepsy, and malignant tumors.

### 2.3 Examination methods

Positional nystagmus was observed and recorded using video nystagmography (Otometrics ICS Chartr 200) in the state of fixation elimination. The position test, vHIT examination, and repositioning maneuvers for BPPV treatment were all performed on the same day.

The vHIT examination was performed using a video head pulse device (Otometrics ICS Impulse 4.0). At least 15 effective flicks were recorded for each semicircular canal. Both the head and eye movement curves were recorded; and the gain value, gain asymmetry (GA), peak saccade velocity, and corrective saccades (CS) were calculated using the software (17). The ratio of GA was calculated as [1-lower gain/higher gain] × 100%. The ratio of CS was calculated as [the number of head impulses with CS]/[trials number] × 100%. The following conditions are considered abnormal in the vHIT: (a) a horizontal semicircular gain <0.8 and a vertical semicircular gain <0.7 (18), accompanied by CS. (b) a normal gain, with CS occurring in more than 50% of trials, i.e., the ratio of CS is more than 50% (19, 20).

All vHIT procedures in this study were performed by senior neurologists specializing in the field of vertigo and dizziness. Data entry was performed using a double-entry system and checked for accuracy.

### 2.4 Statistical methods

The means and distributions were compared using SPSS 26.0 software. Count data were expressed as n/% and compared using a chi-squared test. Normal measurement data were expressed as mean ± standard deviation and compared using a *t-*test. Non-normal data were expressed as M (P_25_, P_75_) and compared using the Mann–Whitney U test for two independent samples. Paired samples were compared using the Wilcoxon signed-rank test. The Kruskal–Wallis test was performed to compare between three groups. The Bonferroni correction was used for pairwise comparisons. Correlation analysis was conducted using ROC curves with MedCalc 22 software. A *p*-value of <0.05 was considered statistically significant.

## 3 Results

### 3.1 General information

A total of 51 cases were included in the light cupula group and 42 cases in the HC-cu group. There was no significant difference in sex, age, and the affected side between the two groups (*p* > 0.05), as shown in Table 1.

**Table 1:** Comparison of sex, age, and the affected side.

	Light cupula	HC-cu	*χ^2^*	*p*-value
*N*	51	42		
Sex (male/female)	9:42	8:34	0.030	0.862
Age	55.39 ± 13.88	52.93 ± 13.08	−1.014	0.311
Affected side (L/R)	26:25	25:17	0.679	0.410

**Figure 1:**
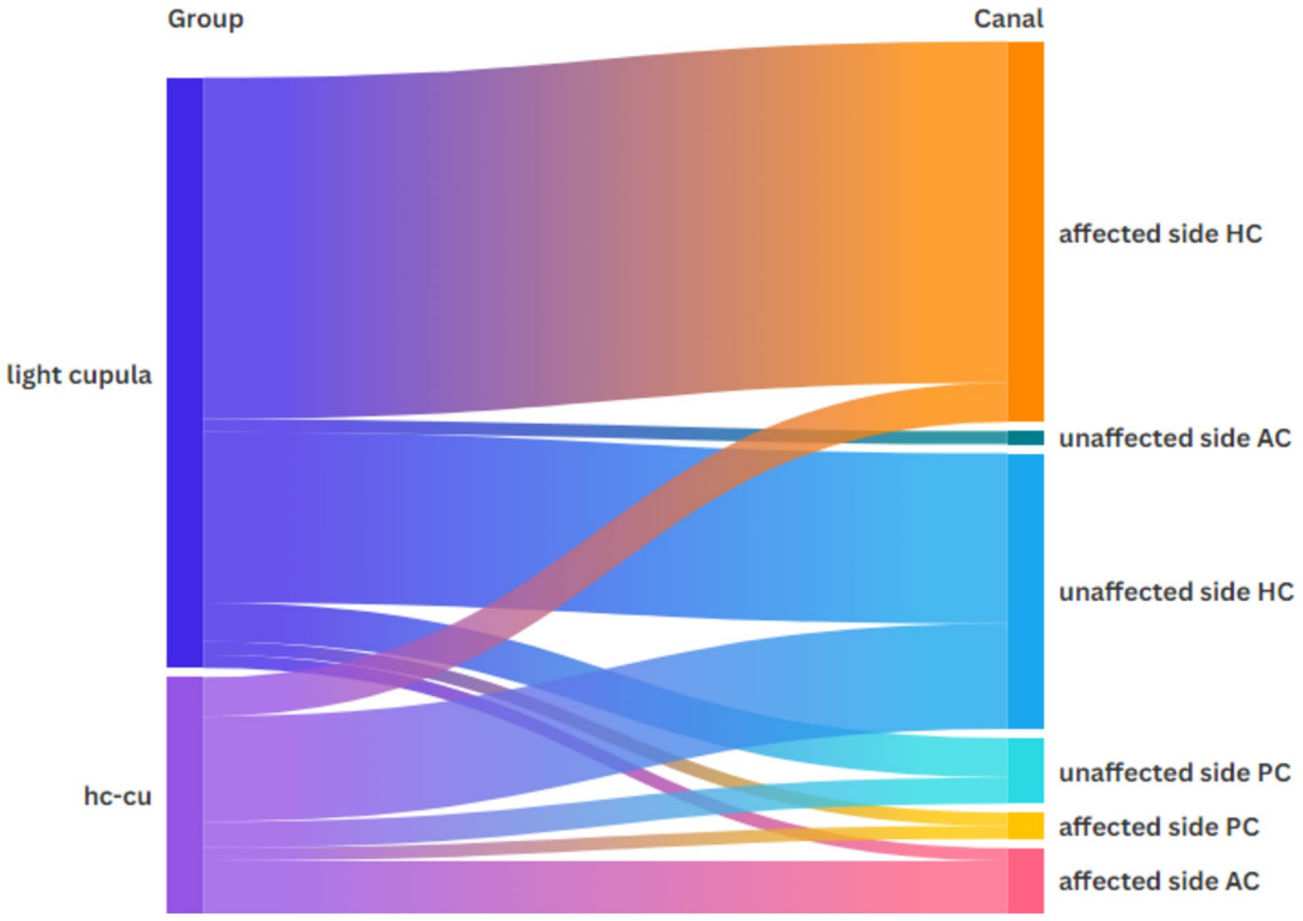
Distribution of the vHIT abnormalities across each semicircular canal in both groups.

Figure 1 shows the distribution of the vHIT abnormalities across the semicircular canals in both groups. From the figure, we can see that the majority of the abnormalities occurred in the horizontal semicircular canal on the affected side of light cupula.

### 3.2 Null plane and positional nystagmus

The Bow-and-Lean test was positive in all cases. PSN was observed in 27 cases (52.9%) of the light cupula group and 14 cases (33.3%) of the HC-cu group. The null plane angles of the light cupula and Hc-cu groups were 30.0 (22.5, 45.0)° and 30.0 (22.4, 45.0)°, respectively. There were no statistical differences between the groups (Table 2).

**Table 2:** Null plane angles and PSN of the light cupula and HC-cu groups.

	Light cupula	HC-cu	*χ^2^*	*p*-value
PSN (presence/absence)	27:24	14:25	2.588	0.108
Null plane (°)	30.0 (22.5, 45.0)	30.0 (22.4, 45.0)	−0.027	0.978

### 3.3 Comparison of the vHIT results

The abnormal rate of the vHIT was 56.7% (29/51) in the light cupula group and 21.3% (9/41) in the HC-cu group, and the difference was statistically significant. Among them, there were 12 cases of abnormal vHIT results on the affected side and normal vHIT results on the healthy side in the light cupula group, while no cases of simple abnormal vHIT results on the affected side were observed in the HC-cu group. The difference was statistically significant. There was no statistically significant difference between the groups in terms of the vHIT abnormalities across different semicircular canals (Table 3).

**Table 3:** Comparison of abnormality detection.

Content of comparison	Light cupula (%) (*n* = 51)	HC-cu (%) (*n* = 42)	*χ^2^*	*p*-value
Abnormal/normal	29 (56.86)	9 (21.43)	11.967	0.001
Affected side only	12 (23.53)	0 (0.00)	–	0.036
Healthy side only	3 (5.88)	4 (9.52)	3.288	0.070
Bilateral	14 (27.45)	5 (11.90)	0.000	1.000
Anterior canal	2 (3.92)	0 (0.00)	–	1.000
Horizontal canal	26 (50.98)	8 (19.05)	0.000	1.000
Posterior canal	4 (7.84)	3 (7.14)	0.687	0.407
Gain reduced	6 (11.76)	4 (9.52)	0.961	0.327

The average gain of the vHIT on the healthy side and the affected side in the two groups was within the normal range, and there was no significant difference between the two groups. The CS% of trials for the HC on the affected side of the light cupula group was higher than that of the HC-cu group, and the difference was statistically significant (see Table 4). The CS% of trials in the light cupula group was higher on the affected side than on the healthy side (the Wilcoxon signed-rank test for the relevant samples, *Z* = −3.675, *p* = 0.00).

**Table 4:** Comparison of the vHIT gain and saccades in the horizontal semicircular canal.

		Light cupula (*n* = 51)	HC-cu (*n* = 43)	*Z*	*p*-value
Gain	Affected side	1.02 (0.93, 1.13)	1.03 (0.95, 1.14)	0.345	0.730
	Healthy side	1.06 (0.94, 1.19)	1.09 (0.94, 1.20)	0.292	0.770
CS% of trials	Affected side	40.0 (13.0, 76.0)	6.0 (0.0, 15.0)	−4.381	0.000
	Healthy side	11.0 (0.0, 38.0)	10.5 (0.0, 35.0)	0.167	0.868

**Figure 2:**
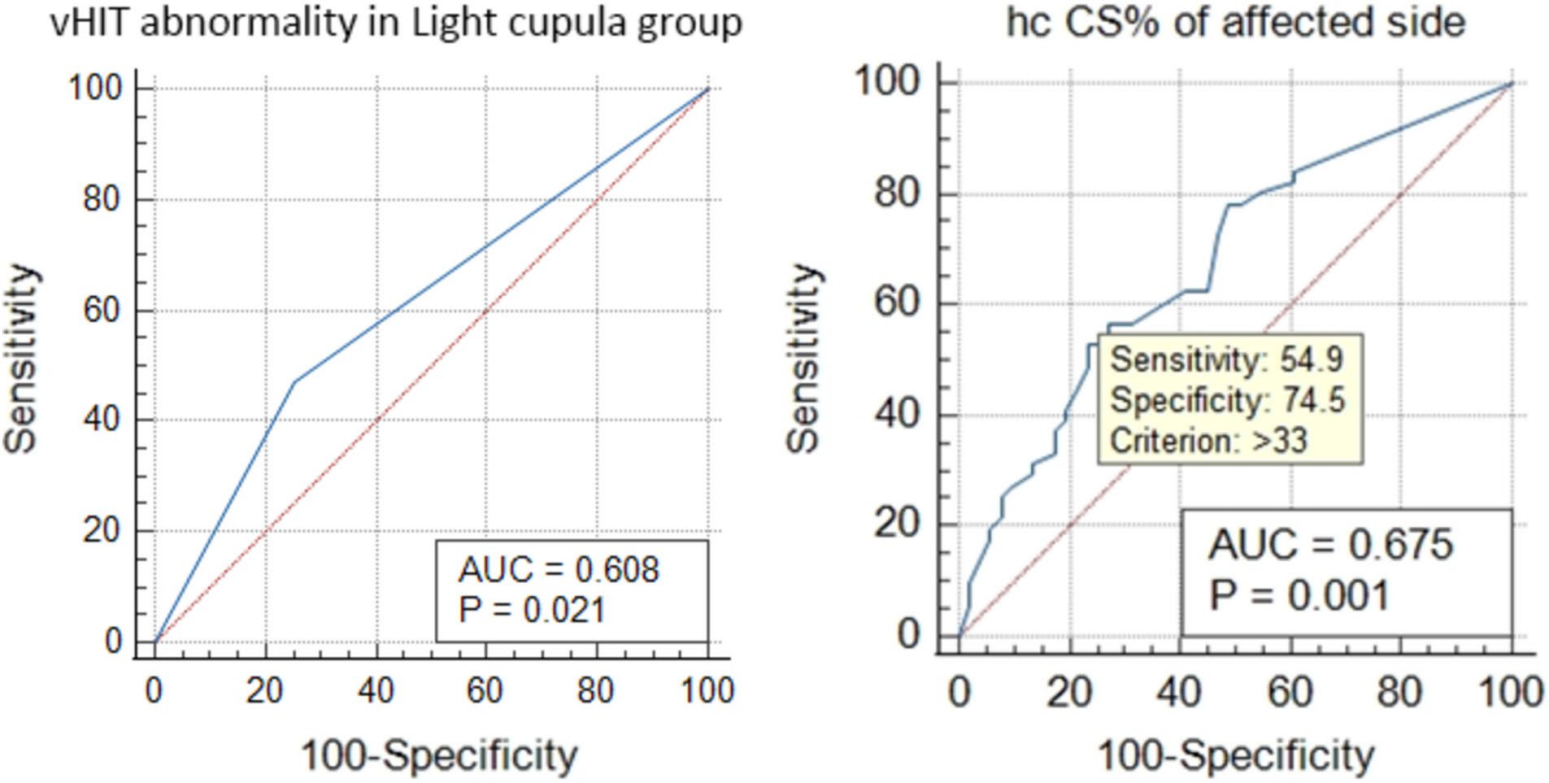
ROC curve analysis of the HC vHIT abnormalities and the correlation of CS% with the affected side in the light cupula group.

The ROC curve analysis showed that the vHIT abnormalities in the light cupula group were correlated with the affected side (AUC = 0.608, *Z* = 2.302, *p* = 0.021), and the ratio of CS was correlated with the affected side (the best cut-off value was 33, AUC = 0.678, *z* = 3.304, *p* = 0.001, sensitivity 54.9%, specificity 74.5%, Figure 2). The vHIT results of the HC-cu group did not show any correlation with the affected side.

## 4 Discussion

The results showed that the vHIT outcomes in the light cupula group were significantly different from those in the HC-cu group. The abnormal vHIT rate in the light cupula group was higher than in the HC-cu group and showed a correlation with the affected side. In this study, the abnormal rate of the vHIT on the affected side was 23.5% in the light cupula group, while no abnormal results were found in the HC-cu group. This finding was consistent with the results of the caloric test, where 21.4% of the light cupula cases showed canal paresis (CP) on the affected side, while no abnormalities were found in the HC-cu group (21). Although the HC-cu group also showed some vHIT abnormalities, these were not correlated with the affected side, which is consistent with the characteristics of BPPV. Numerous previous studies have shown that while there are some scattered vHIT abnormalities in BPPV, they lack a correlation with the responsible semicircular canal (22–26).

Light cupula and HC-cu are often discussed together. They both exhibit opposite persistent DCPN, a null plane where nystagmus disappears, and similar patterns of nystagmus changes in different head positions (8). In this study, there was no difference in the null plane angle, incidence of PSN, or BLT between the two groups. However, their vHIT results were different, suggesting that they are distinct diseases. This finding aligns with the research by Peng H (27).

Not all cases of light cupula showed abnormal vHIT results; 56.9% showed abnormalities, which constituted the majority. The vHIT abnormalities were mainly concentrated in the HC on the affected side, and it could be considered that the vHIT abnormalities were related to the status of light cupula. The abnormal vHIT results mainly manifested as increased saccades, but the gain value did not show a significant decrease. This condition is commonly seen in the recovery phase of acute unilateral vestibular injury (28). Previous studies have suggested that although the gain value may be normal, significantly increased saccades still indicate the impairment of dynamic visual acuity, which is also a manifestation of VOR pathway damage, albeit to a lesser extent (20, 28). In summary, we consider that light cupula may involve a relatively minor degree of VOR pathway injury.

Could the light cupula state itself cause vHIT abnormalities? In the light cupula state, the crista ampullaris is affected by gravity. However, during the vHIT examination of the HC, the patient’s head is upright, and the plane where the HC is located is perpendicular to the direction of gravity, meaning that the crista ampullaris will hardly be affected by gravity. Previous studies have reported that otolith particles floating in the semicircular canals may cause abnormal vHIT outcomes (29–31); however, cupulolithiasis does not have the same effect. The crista ampullaris of HC-cu is also sensitive to gravity, but their vHIT results showed no abnormalities associated with the affected side. Previous studies on posterior semicircular canal cupulolithiasis (PC-cu) have shown (26) that abnormal vHIT results are not related to PC-cu localization. Therefore, we can assume that the light cupula state itself is not the cause of the abnormal vHIT results.

Therefore, there may be some anomalies in the horizontal VOR pathway in light cupula cases, which could be related to the occurrence of light cupula. Seo T (32) reported a case of persistent light cupula secondary to sudden deafness, which is considered to be associated with the irreversible damage of light cupula. In other words, pathological changes in the crista ampullaris itself can lead to both the development of light cupula and injury to the VOR pathway. We speculate that a similar mechanism may exist in idiopathic light cupula, but with a lesser degree of concomitant VOR injury. Among the existing hypotheses, the lighter cupula theory (32) is the one most closely aligned with the results of this study. Due to certain lesions in the crista ampullaris, its density is reduced and its response to high-frequency VOR is decreased, resulting in the light cupula status and abnormal vHIT results.

Among the existing light cupula mechanism hypotheses, the lighter cupula theory (32) and light debris theory (8, 33–35) can independently affect the semicircular canals. The heavier endolymph theory (10–12) and the density difference between perilymph and endolymph theory (36) must consider the possibility of simultaneous involvement of the vertical semicircular canal. The utricular macula theory (37) does not address the issues related to the semicircular canals.

The light debris theory is the hypothesis most similar to the mechanism of cupulolithiasis. However, in previous studies, light cupula was treated with anti-gravity repositioning maneuvers without any success (7, 38), which raises doubts about the validity of the hypothesis of the light debris theory. It is also inadequate for explaining the abnormal results of the vHIT.

Previous studies have almost exclusively focused on the light cupula phenomenon of the HC. However, it is known that the light cupula phenomenon in alcoholic nystagmus occurs simultaneously in all semicircular canals (39). Cases of light cupula involving all semicircular canals on one side, without alcohol intake, have also been reported (40). Positional nystagmus produced by the vertical semicircular canal may be partially counteracted (5, 40). Moreover, the specific angle of the long axis of the cupula of the vertical semicircular canal still lacks anatomical evidence. Therefore, it is difficult to accurately observe and discuss the light cupula phenomenon of the vertical semicircular canal.

Cases of nystagmus with vertical and rotational components were excluded as much as possible. The vHIT results showed that these cases had the highest abnormal rate in the HC on the affected side, confirming that light cupula can occur in the unilateral HC alone. This situation challenges the three hypotheses of the heavier endolymph theory, the density difference between perilymph and endolymph theory, and the utricular macula theory.

It is important to note that the light cupula phenomenon involving all semicircular canals on the same side at the same time does not conflict with the light cupula phenomenon involving a single semicircular canal, and they may have different mechanisms. Similarly, the heavy cupula of the HC is not the same as HC-cu, which is why we named the control group “HC-cu” rather than “heavy cupula.” The light–heavy cupula transformation in alcoholic nystagmus is well known, and similar transformations in non-alcoholic nystagmus have also been reported (13, 41). The heavy cupula may have other mechanisms similar to those of the light cupula, which are not entirely caused by cupulolithiasis. These cases may have been misidentified for a long time, leading to difficulties in identifying non-reducible HC-cu cases. This confusion could help explain the low success rate of HC-cu reduction (3, 27). Therefore, different ideas and considerations should be taken into account in the future exploration of treatment for this condition.

## 5 Conclusion

Idiopathic HC light cupula exhibits significantly more vHIT anomalies than HC-Cu and shows an association with the affected side, while HC-Cu does not display this feature, indicating that the two diseases are different.

The abnormal vHIT findings in light cupula of the idiopathic HC are correlated with the affected semicircular canal, which suggests that light cupula may have a causal relationship or homology with certain injuries in the VOR pathway. The lighter cupula theory is the possible mechanism. The pathological changes in the cupula itself may lead to both the occurrence of the light cupula state and the abnormal results of the vHIT.

Light cupula can affect the HC alone or all three semicircular canals on the same side simultaneously. They may have different mechanisms. These differences should also be considered in future therapeutic explorations.

## Data Availability

The raw data supporting the conclusions of this article will be made available by the authors, without undue reservation.
